# Seeing a Mycobacterium-Infected Cell in Nanoscale 3D: Correlative Imaging by Light Microscopy and FIB/SEM Tomography

**DOI:** 10.1371/journal.pone.0134644

**Published:** 2015-09-25

**Authors:** Marianne Sandvold Beckwith, Kai Sandvold Beckwith, Pawel Sikorski, Nan Tostrup Skogaker, Trude Helen Flo, Øyvind Halaas

**Affiliations:** 1 Centre of Molecular Inflammation Research, Norwegian University of Science and Technology (NTNU), Trondheim, Norway; 2 Department of Cancer Research and Molecular Medicine, NTNU, Trondheim, Norway; 3 Department of Physics, NTNU, Trondheim, Norway; 4 Department of Laboratory Medicine, Children’s and Women’s Health, NTNU, Trondheim, Norway; IPBS, FRANCE

## Abstract

Mycobacteria pose a threat to the world health today, with pathogenic and opportunistic bacteria causing tuberculosis and non-tuberculous disease in large parts of the population. Much is still unknown about the interplay between bacteria and host during infection and disease, and more research is needed to meet the challenge of drug resistance and inefficient vaccines. This work establishes a reliable and reproducible method for performing correlative imaging of human macrophages infected with mycobacteria at an ultra-high resolution and in 3D. Focused Ion Beam/Scanning Electron Microscopy (FIB/SEM) tomography is applied, together with confocal fluorescence microscopy for localization of appropriately infected cells. The method is based on an Aclar poly(chloro-tri-fluoro)ethylene substrate, micropatterned into an advantageous geometry by a simple thermomoulding process. The platform increases the throughput and quality of FIB/SEM tomography analyses, and was successfully applied to detail the intracellular environment of a whole mycobacterium-infected macrophage in 3D.

## Introduction

*Mycobacterium tuberculosis* is the causative agent of tuberculosis, which is the second most deadly infectious disease in the world today [1]. Nontuberculous mycobacteria such as *M*. *avium* cause serious infections in immunocompromised, and sometimes healthy, individuals [2, 3]. Inefficient vaccines and long treatment together with emerging drug resistance demand new strategies to fight mycobacterial diseases [4, 5].

Upon host entry, mycobacteria are phagocytosed by macrophages and dendritic cells. Within macrophages, pathogenic mycobacteria are able to avoid being killed by blocking phagosomal maturation and fusion with the lysosomes [6, 7], yet still retaining the ability for nutritional acquisition [8, 9]. Some mycobacteria are in contact with or even enter the cytosol, becoming targets for autophagy [10–16]. The details and significance of these and similar events are still unknown, and we believe that direct observations of mycobacterium localization and trafficking relative to the host cell compartments could improve the understanding of mycobacterial virulence, survival and killing, and thus contribute in the search towards novel treatment strategies.

High-resolution 3D imaging is required to fully detail the intracellular habitat of the mycobacteria, but many cellular structures of interest are beyond the resolution of light microscopes. Focused Ion Beam/Scanning Electron Microscopy (FIB/SEM) tomography is a valuable and little explored alternative, with significantly reduced manual labor compared to non-automated 3D electron microscopy (EM) techniques. During FIB/SEM tomography, a dualbeam instrument comprising a FIB and SEM is used to successively mill away thin slices of material (typically 10-100nm thick) from a sample block, and image the appearing sample surface with the scanning electron beam. This results in an ultra-high resolution image stack representing the cell or region of interest in three dimensions.

To increase the specificity of FIB/SEM studies, initial selection of a region of interest and imaging of labeled structures can be performed in an optical fluorescent microscope. If the very same region can be relocated and imaged at the EM level, this is called correlative imaging. Correlative imaging studies with light microscopy (LM) and FIB/SEM have been performed [17–20], but most systems for correlative light and electron microscopy (CLEM) have been optimized for imaging on ultrathin sections with TEM [21–25]. In contrast to TEM, FIB/SEM tomography is an *en bloc* technique, and thus requires a different approach, especially when it comes to LM and EM imaging and correlation between the two. Due to the geometry of the FIB/SEM instrument, the throughput of FIB/SEM tomography experiments can be increased if regions of interest are located close to a sample edge rather than on a flat surface, avoiding the need for trench milling around the region of interest [26]. This would also reduce sample damage from ion beam exposure, and increase the volume readily accessible during FIB/SEM tomography experiments by reducing shadow and charge effects.

To meet the need for a 3D CLEM platform optimized for LM and FIB/SEM tomography, we developed a reproducible system allowing targeted high throughput and high quality FIB/SEM tomography studies of adherent cells such as macrophages. The presented system is based on a micropatterned Aclar substrate, on which adherent cells are cultured within microwells of a predefined size. The cells can be studied with light microscopy within the wells, and an integrated reference system ensures proper cell localization. After contrasting for EM and plastic embedding, the aclar substrate is removed, leaving cells embedded in small protruding epoxy blocks. We show the successful use of this system for investigation of mycobacterium-infected primary human macrophages. Cells imaged using confocal microscopy were easily relocated for 3D imaging with FIB/SEM, allowing identification of the labeled structures with significantly enhanced resolution. The sample geometry allowed for straightforward overlay between data sets from confocal and FIB/SEM, with minimum manual adjustments. Important cellular structures such as mycobacterium-containing phagosomes were accurately resolved during FIB/SEM imaging. The presented system is simple to produce and use, and increases the quality, specificity and throughput of correlative 3D confocal fluorescence microscopy and FIB/SEM tomography studies.

## Results

Biological samples are fixed, dehydrated, stained and embedded in a plastic block (e.g. epoxy) before imaging is performed in a FIB/SEM instrument. A system for correlative imaging of adherent cells must therefore consist of a platform where light microscopy imaging and localization of cells can be performed, and where both imaged cells and the reference system used for localization can be transferred to an epoxy block. The following sections describe the production and performance of such a substrate for 3D CLEM, where the optimal geometry for FIB/SEM tomography has been taken into account (S1 Fig). The substrate is based on thermomoulded Aclar, a transparent poly(chloro-tri-fluoro)ethylene film often used for CLEM studies of cellular monolayers due to its compatibility with cell growth, light and fluorescence microscopy and EM sample preparation methods for biological samples [18, 27, 28].

### Production of aclar microwell substrates for cell growth and imaging

**Fig 1:**
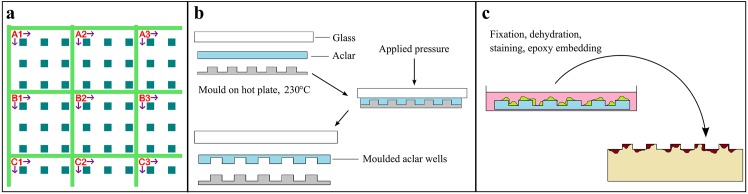
Master design and aclar substrate production. A silicon master was designed and produced in order to pattern aclar films by thermomoulding. a) The master design, including an array of 100 μm sized squares and a reference system for simple localization of cells with various imaging techniques. b) Micropatterned aclar substrates were produced from the master by thermomoulding. The master was heated to 230°C, before a piece of aclar and a glass microscopy slide was placed on top. An even pressure was applied to the assembly, before subsequently cooling down and separating the individual components. After thermomoulding, the microblocks from the master became wells in the aclar substrate. c) Illustration of how primary human macrophages could be cultured in the wells. After *M*. *avium* infection and confocal imaging, cells were fixed, stained, dehydrated and embedded in epoxy for FIB/SEM tomography. The aclar substrate was removed after resin polymerization, creating an array of protruding microblocks containing immobilized cells.

To create a substrate suitable for cell growth, precise localization of cells and correlative light and FIB/SEM imaging, an array of microwells arranged in a reference system was imprinted into aclar by thermomoulding (Fig 1). A silicon master of regularly spaced 100 μm *×* 100 μm *×* 20 μm microblocks superimposed on a 3μm high reference grid was produced by a combination of UV- and electron-beam lithography and two-step etching (see Materials and Methods). The pattern from the silicon master was transferred into aclar by a simple thermomoulding process, where the polymer film was pressed against the heated silicon mould (see Materials and Methods) (Fig 1B). The process allowed patterning of areas suitable for cell experiments (typically ~1 cm^2^ was used in this work). The pattern was transferred with high fidelity, preserving features down to sub micrometer scale (S2 Fig). The transfer process was equally reliable at the step from aclar to epoxy, allowing the initial design of the master to be replicated at this final step.

### Light microscopy and FIB/SEM tomography of mycobacterium infected macrophages

**Fig 2:**
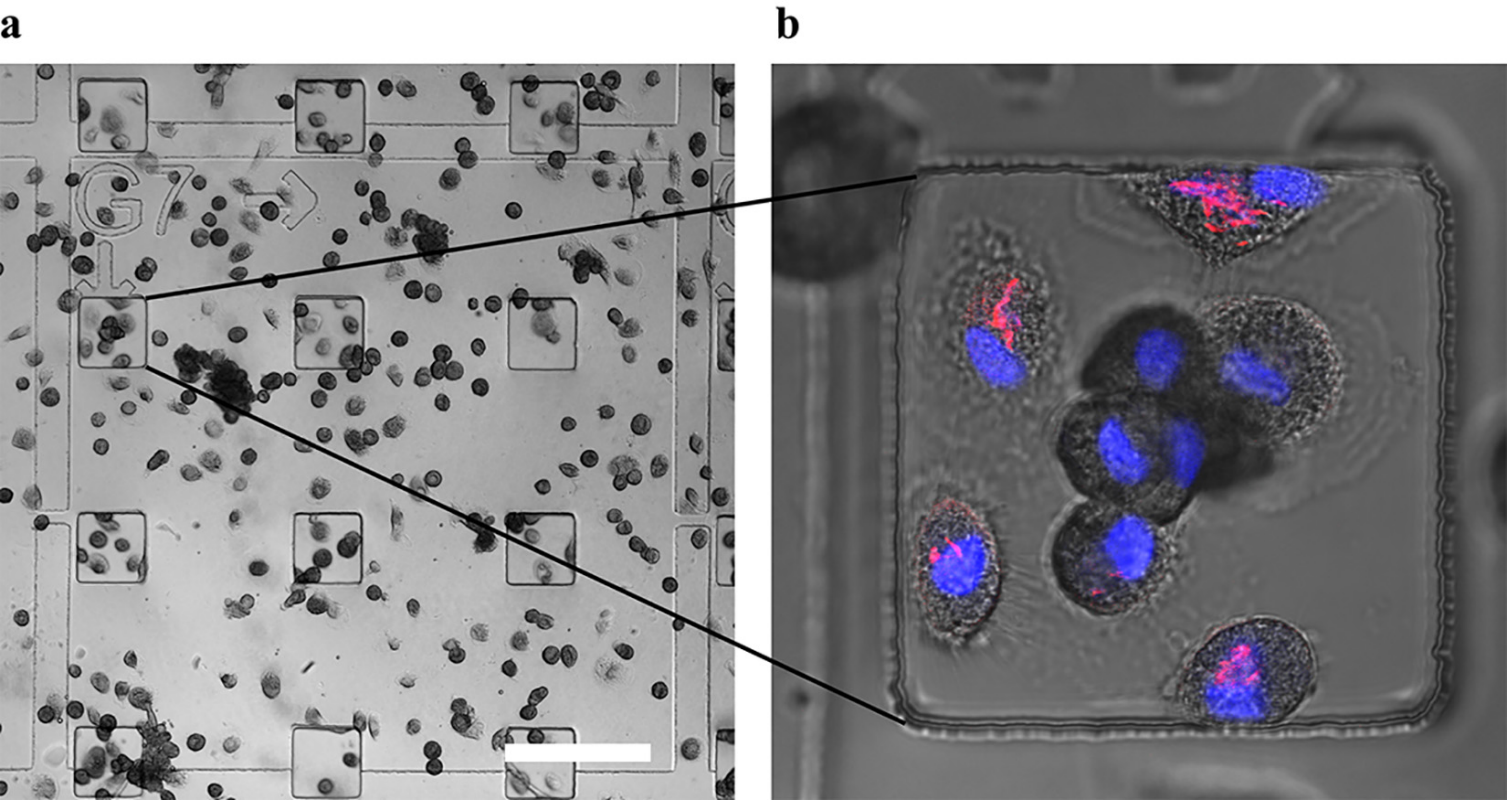
Light microscopy. Primary human macrophages were grown and infected with CFP-expressing *M*. *avium* on aclar microwell substrates. The cells were then aldehyde fixed and fluorescence stained for nucleus, before light microscopy imaging was performed. a) DIC image illustrating the excellent visibility of both microwells and reference system that were imprinted in aclar by thermomoulding. b) Confocal fluorescence microscopy image with DIC overlay, displaying nuclei in blue and bacteria in red. About 70% of infected macrophages contained at least one bacterium (n = 100). Scale bar 200μm.

**Fig 3:**
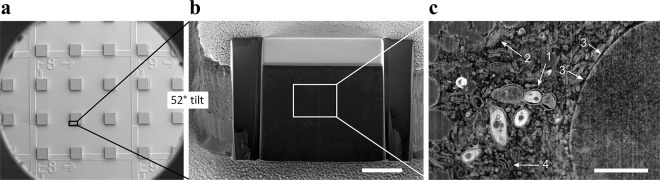
FIB/SEM imaging. After confocal imaging, *M*. *avium*-infected primary human macrophages were dehydrated, stained and embedded in epoxy. a) SEM micrograph of the final epoxy sample surface after removal of the aclar substrate. The topography of the reference system is sufficient to remain visible at this level. All cells located at an edge of a small protruding block are immediately accessible for FIB/SEM tomography analyses. b) After localization of a region of interest (indicated by the black rectangle in a), the sample was tilted to 52° and the cell of interest was exposed by ion-beam milling. c). SEM micrograph collected during a FIB/SEM tomography experiment, from the surface area indicated by a white rectangle in b). White arrows indicate the following observable details: 1: mycobacteria surrounded by phagosomal membranes, 2: Golgi, 3: nuclear pores recognized by discontinuities in the double nuclear membrane, 4: mitochondrion. Scale bars: 10 μm in b, 2μm in c. The blocks in a) are about 100μm wide.

Monocytes isolated from healthy human blood donors could be reproducibly grown and differentiated into macrophages by adherence to the micropatterned aclar substrates (Fig 2). The aclar well size of 100 μm *×* 100 μm was suitable to accommodate macrophages, which routinely stretched out more than 50 μm. The repeating pattern of closely spaced microwells assured that a large number of cells grew close to or attached to one of the well edges. This resulted in a large number of cells located on straight sample edges after epoxy embedding, all of which are particularly well suited for FIB/SEM tomography analysis (Fig 3). The number of cells per well was typically between five and ten.

Macrophages were examined minimum two days post infection with *M*. *avium* expressing cyan fluorescent protein (CFP), to address viable mycobacteria having escaped the initial destructive phase. Infected cells could be fixed and stained for cellular components, without detachment from the aclar substrate. For confocal imaging, aclar substrates were placed upside down in a glass-bottomed dish suitable for confocal imaging. Light microscopy was performed on fixed cells in buffer, and was usually best with a water objective due to a thin liquid layer present between the cells and the glass bottom. Both microwells and the reference grid were clearly visible with wide field light microscopy, and z-stacks of stained cells and CFP-expressing bacteria could be gathered by confocal fluorescence imaging (Fig 2). After confocal imaging, the cells were dehydrated, contrasted for FIB/SEM and infiltrated with epoxy resin (see Materials and Methods). After epoxy polymerization, the aclar substrate was peeled off, and immobilized cells remained in small epoxy blocks protruding upwards from a flat surface (Fig 3A). The separation process of aclar from cured epoxy was reliable, with no cells remaining attached to the aclar after separation. Cells of interest in microwells identified by confocal microscopy before embedding could easily be localized in corresponding protruding epoxy blocks in the FIB/SEM instrument by using the reference grid that remained visible after transfer to epoxy (Fig 3A). Regions of interest within single wells were further located by measuring the distance from the well/block edge both on confocal images and on secondary electron images. During SEM imaging, a high acceleration voltage of the electron beam (15kV- 25kV) allowed observation of the shape of the underlying cells due to increasing electron penetration depth with acceleration voltage (S3 Fig). This further facilitated a correct placement of the region to be investigated in correlation with the confocal images gathered earlier.

After localization of the exact region of interest, the region was prepared for FIB/SEM tomography (Fig 3B), and slice and view data was collected (see Materials and Methods). In the collection of 3D FIB/SEM data, the slice thickness was chosen at 35nm. Each electron microscopy image covered an area of about 20 μm x 20 μm, resulting in a pixel size of about 10nm. Despite the relatively large pixel size resulting from the large field of view, SEM images were of a quality comparable with TEM images, with sufficient contrast and resolution to discern features such as mycobacterium-containing phagosomes, mitochondrial and Golgi cisternae, the double nuclear membrane and nuclear pore complexes (Fig 3C, see S4 Fig for a more detailed comparison). Similar features were poorly resolved in cells prepared as a pellet, due to insufficient penetration of the contrast agent into the block (data not shown). The increase in contrast for cells prepared as monolayers compared to cells prepared as pellets is likely due to a drastic decrease in sample thickness for cellular monolayers, resulting in improved contrast agent penetration.

### Volume correlation and 3D reconstruction

**Fig 4:**
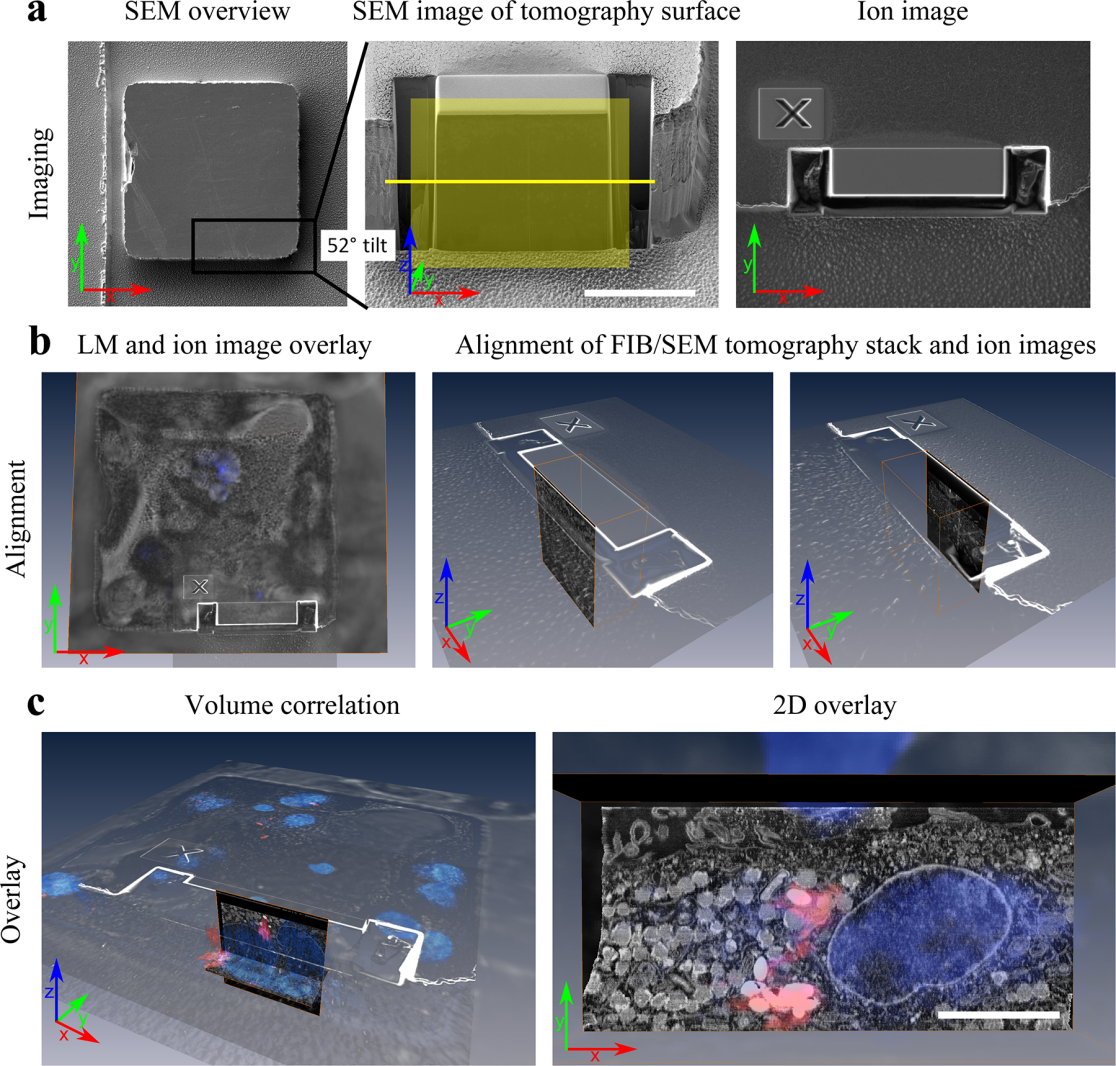
Volume correlation procedure. One entire microwell was imaged by light microscopy (DIC and confocal fluorescence microscopy), while one cell at an edge of the same well was imaged by FIB/SEM tomography. a) Overview SEM image of the well of interest after epoxy embedding, and SEM image of the region of interest at the corner of the well after preparing the region for FIB/SEM tomography. The images collected during the tomography experiment are in the x-z plane (yellow square), while the images collected during LM imaging are in the x-y plane, parallel to the well surface (yellow line). Ion images are collected in the x-y plane before each milling step, where the cross serves as a reference marker enabling each slice to be milled with nanometer precision. b) Illustration of how the shape of the microwells can be used to place the volume imaged during FIB/SEM tomography correctly within the larger volume imaged during LM imaging. The ion images are first aligned with the edges of the well as imaged by LM. The perpendicular FIB/SEM tomography images can then be aligned with the ion images, with the first and the last image overlapping with the edge observed in the first and last ion image. c) After alignment the two volumes are correlated, and an overlay between fluorescence and EM images can be done in any plane or volume. In the 2D overlay, an xy projection from the FIB/SEM stack is overlaid on the corresponding fluorescence image, with nuclei displayed in blue and bacteria in red. Scale bars: a: 20μm, c: 5μm.

As illustrated in Fig 4A, the imaging planes from confocal and FIB/SEM imaging are perpendicular to each other. A unified coordinate system for both light and electron images was defined by designating the confocal imaging plane as the x-y plane, and the FIB/SEM tomography imaging plane as the x-z plane. To overlay the 3D datasets of the cells obtained by confocal microscopy and FIB/SEM tomography, an alignment procedure based on distinct geometrical features of the sample (such as well edges) was implemented using Avizo. Briefly, the confocal dataset was first aligned in the x-y plane to ion beam images acquired simultaneously with the tomography data during the FIB/SEM tomography experiment (Fig 4B). Further, the relative z-positions were aligned by identifying the confocal slice at the base of the well from differential interference contrast (DIC) image sections of the confocal stack. The first and last images of the SEM image stack resulting from the FIB/SEM tomography experiment were then aligned to the edges of the tomography volume swept by the ion images (Fig 4B). Finally, an overview image from the block face prepared for FIB/SEM tomography was used to overlay the SEM image stack to the ion images in the x-z plane. In this way, correlation between the SEM stack and confocal microscopy stack was performed purely on the basis of sample geometry, allowing accurate 2D and 3D correlation and overlays between light and electron microscopy data (Fig 4C).

**Fig 5:**
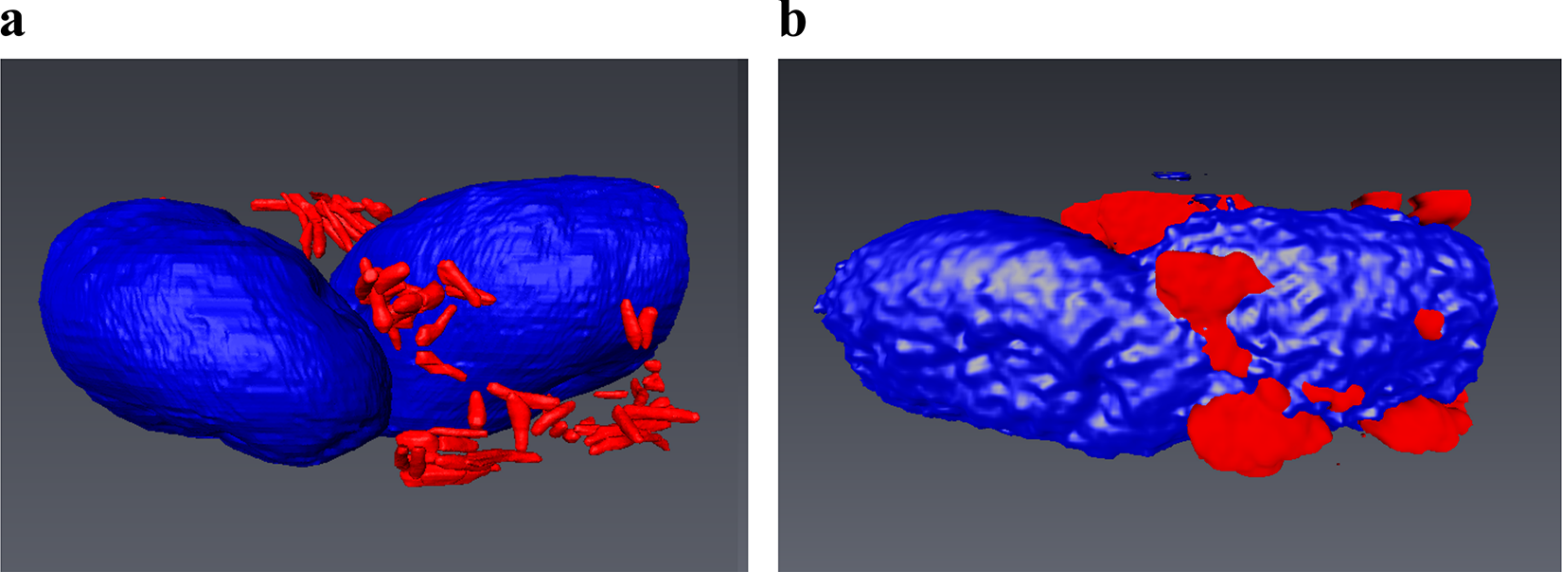
3D reconstruction after correlative imaging. a) and b) 3D models of the same cell, reconstructed from a FIB/SEM tomography stack and a confocal z stack respectively. The nuclei are displayed in blue and mycobacteria in red. The correspondence between the two models is good.

**Fig 6:**
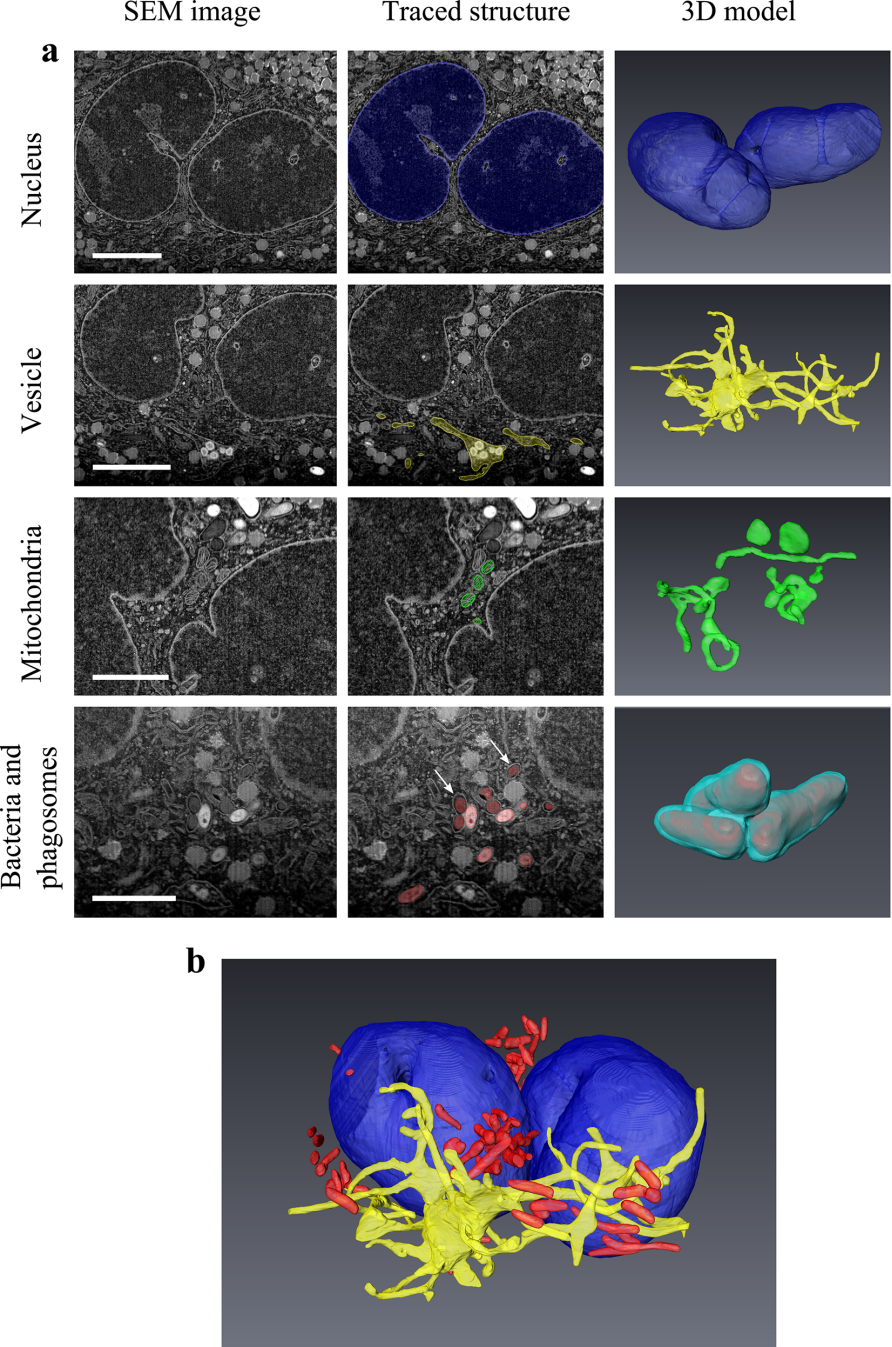
3D models. Selected structures were reconstructed in 3D from the image stack obtained during a FIB/SEM tomography experiment. a) Column 1 shows a representative SEM image from the original stack while column two outlines how the segmentation was performed on these images to create the 3D models shown in column 3. Nuclei are colored in blue, mitochondria in green, a continuous vesicular structure in yellow, bacteria in red and a selected phagosome in cyan. In row 4, white arrows point to two phagosomal membranes surrounding. Similar membranes were traced to reconstruct the phagosome enveloping 3 bacteria in the lower right Fig b) 3D model including nuclei, vesicle and bacteria. Scale bars: Nucleus and vesicle: 5μm, Mitochondria and bacteria/phagosomes: 3μm.

3D models of an *M*. *avium* infected macrophage were created from the image stacks collected with both confocal and FIB/SEM techniques, using Avizo. To create 3D models from the FIB/SEM tomography image stack, contours with increased contrast (typically lipid membranes) of structures of interest were manually traced on each image of the stack. A surface was generated from the traced contours by interpolating over the known distance between each image (i.e. 35 nm in this case) throughout the whole stack (Figs 5A and 6). Clearly resolved membranes were used to determine boundaries and internal structures of organelles, bacteria and phagosomes. The process of generating surfaces from the FIB/SEM stack is also illustrated in S1 Video. 3D surfaces were rendered from the confocal image stack was surface rendered by a threshold algorithm.

Fig 5A and 5B demonstrate corresponding confocal and FIB/SEM 3D volume reconstruction results of the nucleus and bacteria in an *M*. *avium* infected macrophage. The models derived from confocal and SEM imaging data correspond well in terms of the shape of the nucleus and the localization of the bacteria, confirming that the system is well suited for performing targeted FIB/SEM studies after selecting cells of interest by confocal imaging. In the 2D overlay displayed in Fig 4C, not all fluorescent areas from the confocal image correspond precisely to features in the SEM image. However, all features correspond if the datasets are visualized in 3D as illustrated by the models in Fig 5A and 5B. This highlights the difference in resolution of the two methods, where the fluorescent signal of one image in the confocal stack is collected from a much larger volume (optical slice thickness of several hundred nm) than the signal of one SEM image from the FIB/SEM stack (a few nm).

### 3D rendered model portray intracellular environment of *M*. *avium*-infected macrophage at high resolution

The electron image data obtained by FIB/SEM tomography together with corresponding 3D rendered models allowed further elaboration of the intracellular environment of mycobacteria in macrophages during an established infection (Fig 6). Reconstruction of the nucleus revealed that the cell of interest contained two separate nuclei, both pierced by several bifurcated tunnels (Fig 6A). The trans-nuclear tunnels contained cytoplasmic components such as mitochondria, strongly suggesting that these structures were not artifacts from sample preparation. The trans-nuclear tunnels are consistent with earlier descriptions of nucleoplasmic reticula [29, 30]. Nucleoli could be identified as bright regions within both nuclei, and nuclear pores and the double nuclear membrane could be discerned. Mitochondria showed a large variability in size and shape, as illustrated by 3D models of selected entities (Fig 6A).

Importantly, mycobacteria were easily identifiable by their strong contrast and distinct slightly ellipsoid cross-sections, aided by the confocal overlays of the fluorescent bacteria. The bacteria were often observed in clusters surrounded by phagosomal membranes which were traced and reconstructed in 3D, discerning neighboring mycobacteria in separate compartments as well as multiple mycobacteria within the same phagosome (Fig 6A). The interconnection of the phagosomes was only visible after reconstruction in 3D of the SEM images, and would thus be difficult to confirm in standard TEM experiments on selected thin sections. This highlights the importance of high-resolution 3D imaging of large volumes for proper characterization of an intracellular environment. Similarly, single images displayed a number of seemingly separate vesicles, but these were revealed as a single large tubulovesicular structure after 3D reconstruction through the complete image stack (Fig 6A). This structure further contained internal vesicles and structures, reminiscent of a multivesicular body or a tubular lysosomal compartment. The relation between reconstructed structures could be observed when 3D models of various components were combined, giving a greater understanding of spatial relations between intracellular compartments (Fig 6B and S1 Video).

## Discussion

We report the use of FIB/SEM tomography correlated with confocal fluorescence microscopy to image primary human macrophages infected with mycobacteria. FIB/SEM tomography enables imaging of whole cells at an ultra-high resolution and in 3D, while light microscopy can identify specific fluorescently labelled structures. Correlation of the two techniques allows to map labelled structures in the broader structural and cellular context offered by FIB/SEM tomography. Also, since primary human macrophages differ in morphology and the infection efficiency with mycobacteria varies greatly, the success rate of FIB/SEM tomography in these experiments could be increased if initial selection of cells of interest is performed at the LM level. A challenge is that current CLEM systems have been optimized for the use of TEM and not FIB/SEM applications. FIB/SEM samples need to be contrasted *en bloc* and embedded in plastic before imaging, and volumes of interest should be located on a sample edge rather than on a flat surface. One group has reported on correlative imaging of adherent cells using FIB/SEM tomography, but the sample geometry was then not taken into account [18].

In order to create a system of optimal geometry for FIB/SEM tomography experiments, while retaining compatibility with both light and electron microscopy preparation procedures and imaging, we chose to base our platform on the polymer film aclar. Aclar allows growth and preparation of adherent cells (such as macrophages) for EM, compatibility with EM-chemicals, and convenient separation of the substrate from the epoxy-embedded cells after resin curing. To create a substrate of the desired geometry, we exploited a well-known but little used characteristic of aclar, namely its thermoformability. Bunge et al. used this method to create custom shaped cell culture dishes already in 1973 [31]. Here, we extended thermoforming into the micron scale. To create well-defined and durable silicon masters, standard micro fabrication processes were applied. Once the master was created, a large number of substrates could be thermomoulded using standard lab equipment. The straight-edged well/block geometry presented here assures that a large number of cells were located on sample edges, thus drastically increasing the number of cells readily accessible for FIB/SEM tomography. As the lithographic patterning of the master is highly versatile, different well/block geometries or reference patterns are easily within reach. For instance, microblocks with a suitable size and shape for TEM sectioning could be produced.

The aclar substrates supported differentiation and growth of primary human macrophages, and long-term infection with *M*. *avium*. Here the infected cells were imaged with a confocal microscope after chemical fixation, but as other groups have shown, aclar is also compatible with live cell imaging and subsequent high-pressure freezing and freeze substitution [28, 32], with the possibility of preservation of fast temporal events. This would also reduce autofluorescence from glutaraldehyde that decreased the quality of confocal images in our experiments [33]. It should be noted however, that in this work confocal imaging was performed on a large area (125 μm *×* 125 μm) to scan a large number of cells for the presence of bacteria. Although not the focus of this work, fixation and imaging procedures could be optimized for higher resolution fluorescence imaging of single cells if desired, which would presumably enhance the detail of the resulting overlay data.

In particular, modern developments in superresolution fluorescence imaging has pushed the resolution of light microscopy towards the realm of electron microscopy. Single molecule localization techniques such as PALM and STORM have demonstrated 10–20 nm lateral and axial resolution of labelled proteins [34]. Localization microscopy benefit strongly from correlative imaging approaches, and indeed, several variations of correlative superresolution microscopy and electron microscopy have been reported [35, 36]. Superresolution optical microscopy would contribute to the investigations of the intracellular life of *M*. *avium* bacteria in macrophages by providing further details of protein localization and potential interactions. However, it remains to be investigated if the aclar substrates developed here are suitable for superresolution imaging.

After EM sample preparation and embedding in epoxy, the convenient reference grid allowed localization of cells imaged by confocal microscopy in the FIB/SEM instrument. This allowed both initial screening of interesting cells and later correlation of specific confocal data with ultrastructural FIB/SEM data. Volume correlation between the datasets collected by confocal fluorescence microscopy and FIB/SEM tomography was performed based only on the geometrical features present in the moulded aclar substrate. In contrast to methods involving correlation based on biological features or added reference particles [19] the method presented here allows greater flexibility in terms of sample preparation and labeling. Further, possible biases introduced by using sample data for both alignment and analysis are avoided.

In our system, the accuracy of the correlation procedure was limited by the accuracy of the overlay between ion images of the well replica acquired during the tomography experiment, and DIC images of the edges and base of the aclar well collected in parallel with the confocal data set. The overlay accuracy could thus be further improved by using smaller, easily identified patterns at the bottom of the wells or the rest of the substrate, that could be included in the initial silicon stamp. Optimally, the accuracy of the volume correlation is in the order of the voxel size of the DIC/confocal data set.

There are also certain inherent limits to the overlay accuracy, imposed by possible changes to the sample after LM imaging, the limited resolution obtainable by LM and the subjective interpretation of the limits of a fluorescent signal (e.g. the threshold chosen for 3D reconstruction). Dehydration has been reported in the literature to cause tissue shrinkage of various degree [37–39]. Here, a strong fixation protocol was used, which has been shown to result in minimal shrinkage after dehydration [38]. The percentage of shrinkage of our samples was too small to assess by measurements on the respective LM and FIB/SEM data. 2D representations of data overlays from volume techniques will differ from correlative imaging based on thin slices due to the large difference in signal volume and axial resolution between 3D light and electron microscopy, reducing the apparent 2D overlay precision. However, full 3D imaging of comparatively large volumes and overlay of resulting 3D data offer great advantages that in many cases outweigh the reduced overlay precision of single 2D images from the datasets.

FIB/SEM tomography has been demonstrated on several biological systems [17, 40–46], but the technique is still new and under development. TEM techniques have had a more widespread use, including important contributions revealing the phagosomal escape of strains of pathogenic mycobacteria like *M*. *tuberculosis* into the cytosol [11, 15]. TEM is indeed a powerful technique, but the third dimension added by FIB/SEM tomography is central for certain studies. Autophagy is a cellular process of great interest in the context of infection, and intracellular structures such as mitochondria and ER have been confused with autophagosomes on thin sections for TEM [47]. Correlation with LM can further improve studies of autophagy and similar processes where known proteins are involved. Subramaniam and his team used FIB/SEM tomography to reveal new aspects of the immunological synapse between T-cells and dendritic cells during HIV infection, and to reveal conduits in HIV-infected macrophages [40, 41]. Similar ultrastructural 3D investigations would be of interest for mycobacterium infections. FIB/SEM tomography also enables complete identification of the intracellular habitat for all bacterial colonies, as it is unlikely that all bacteria inside an infected cell are equal [48].

In our investigations, FIB/SEM tomography allowed identification of *M*. *avium* bacteria localized by fluorescence microscopy, as well as characterization of their intracellular surroundings within host macrophages with nanometer resolution. *En bloc* staining of macrophage monolayers on aclar gave sufficient contrast to observe the detailed cellular ultrastructure during FIB/SEM tomography experiments, at a resolution comparable to TEM. In one representative cell, no bacteria were observed without a surrounding phagosomal membrane, and single phagosomes often contained several bacteria. Phagosomal membranes were closely spaced around bacteria, corresponding well to descriptions of *M*. *avium*-containing phagosomes observed with TEM [49], and different from the phagosomal escape that has been demonstrated for *M*. *tuberculosis* and *M*. *marinum* [14, 15]. In addition to bacteria and phagosomes, structures such as mitochondria, nuclei and vesicles could be characterized from the same FIB/SEM image stack. Mitochondria of various shapes could be observed after 3D reconstruction, and one large tubulovesicular body that was continuous throughout a significant volume of the cell was identified. The vesicle, which contained additional cellular components, could be the endocytic recycling compartment or a tubular lysosomal compartment [50], however this remains to be verified by CLEM using specific labels for this and other compartments. Interestingly, a clear manifestation of the nucleoplasmic reticulum was apparent after 3D reconstruction. The nucleoplasmic reticulum has only recently been described, but has never been observed in primary human macrophages and has not been imaged in 3D at a comparable resolution [29, 30]. The high resolution and large diversity of observed structures highlights the power of FIB/SEM tomography when compared to confocal fluorescence microscopy, where only specifically labeled molecules or organelles are imaged at a more limited resolution. However, the combination of confocal microscopy and FIB/SEM tomography is even more beneficial than either of the two alone, as intracellular structures can be identified using specific labels and stains visible by confocal imaging and thereafter resolved in high-resolution 3D images along with their surroundings.

In summary, the system developed in the present study constitutes an accessible and easy to use-platform, suitable for streamlined high resolution FIB/SEM investigation of adherent cells. The platform also enables reproducible correlative imaging by high resolution, high numerical aperture confocal fluorescence microscopy and FIB/SEM tomography. Throughout the process, cells are maintained in their adherent state, without resorting to potentially destructive techniques such as scraping or pelleting. Cell growth is performed according to standard protocols, and confocal imaging can be carried out without any modifications to the light microscope. The use of aclar as a growth substrate allows for simple EM sample preparation, including reliable and quick separation of the substrate from cured epoxy. Not only do the wells largely simplify the localization of the exact same cell with several microscopy techniques, but they also constitute a platform that is very advantageous to work with in a FIB/SEM. Straight edges assure good accessibility to many cells for FIB/SEM tomography, and decrease the time required for milling before starting the automatic image acquisition. The correlative imaging platform allowed volume correlation based only on features of the substrate, without bias from cellular components. It was shown to be very useful for inspection of primary human macrophages infected with *M*. *avium*, and the method could be similarly applied to detail the intracellular milieu of any adherent cell in high-resolution 3D.

## Materials and Methods

### Stamp production

The master for molding Aclar poly(chloro-tri-fluoro)ethylene microwells was made using silicon microfabrication processes. The hard mask for etching the wells was patterned using photolithography, while the reference grid was patterned with electron beam lithography, before a two-step cryoetching process was performed to transfer the patterns into the silicon wafer.

A p-type <110> silicon wafer was cleaved into 2x2 cm square pieces. SPR700.1 photoresist (Microchem) was patterned using photolithography with an inversed 100/200 μm well-pattern in a Carl Suss MA6 mask aligner. A hard mask for etching the wells was deposited by first DC-sputtering 15 nm aluminum, and then allowing 10% O_2_ into the chamber for reactive DC-sputtering of 15 nm of Al_2_ O_3_. The sputtering was performed in an AJA Custom ATC-2200V sputter-coater at 300W and 3 mtorr. Lift-off was performed by sonicating for 5 minutes in Remover PG (Microchem).

For producing the reference grid, a double-layer of Omnicoat (Microchem) was applied prior to the SU-8 for simple removal of SU-8 later. A 1 μm SU-8 2 film was patterned by electron beam lithography using a Hitachi S-4300 FEG-SEM modified with a Raith Quantum patterning system, with a voltage of 30 kV, a beam current of 500 pA and a dose of 1 μC cm^*−*2^, leading to a total patterning time of only 20 minutes despite the large area. After development, the exposed areas of Omnicoat were removed by exposure to a 50 W 0.6 mbar oxygen plasma for 1 minute in a plasma cleaner (Diener Femto).

The reference grid was transferred into the silicon substrate by a highly selective and anisotropic SF6 /O_2_ cryogenic etch in an Oxford PlasmaLab 180 ICP-RIE. The etch was performed at *−*120*°*C, with 90 sccm SF_6_, 11.5 sccm O_2_, an RF platen power of 4W, an ICP power of 750 W and a process pressure of 7.5 mtorr, which gave an etch rate of about 1.45 μm min^*−*1^. After 2 minutes of etching, the sample was removed and the remaining SU-8 resist was removed by sonication for 2 minutes in Remover PG at 80*°*C. The sample was then etched for a further 13 minutes with only the alumina hard mask, defining the wells. The deep silicon etching process ensured straight vertical well edges, which is optimal for FIB/SEM milling and imaging

Finally, the aluminum/alumina mask was removed by soaking in MF-26A developer (Microchem) for 2 minutes. The surface was cleaned with a 50 W oxygen plasma for 18s at 0.6 mbar, before the surface was treated with an anti-stick layer of 1H,1H,2H,2H-Perfluorooctyltriethoxysilane (Sigma Aldrich) by vapor silanization for 15 minutes.

### Thermomoulding of aclar

Aclar poly(chloro-tri-fluoro)ethylene films (7.8 mil, Ted Pella) were cleaned by rinsing in ethanol. The silicon master was heated to 230*°*C on a hot plate, and the aclar film was carefully placed on top and allowed to conform to the surface. A 2x2 cm piece of a glass microscopy slide was briefly heated on the hot-plate, and then placed on top. Force was applied on top of the glass with the blunt end of a pair of tweezers until no air bubbles in the aclar could be observed. The master, film and glass stack was removed from the hot-plate and allowed to cool down for 5 minutes, before carefully separating the pieces using a scalpel at the edge. The maximum durability of the master was not tested, but it still performed consistently after patterning at least 50 aclar substrates.

### Cell culture and bacterial infection

Moulded aclar films of size 1x1 cm were sterilized in ethanol, air dried in a sterile bench and placed in 24 well plate flat bottomed culture dishes (Corning Costar) for cell culture.

Human peripheral blood mononuclear cells (PBMCs) were isolated from buffy coats obtained from the Blood Bank, St Olavs Hospital, Trondheim by density gradient centrifugation (Lymphoprep; Axis-Shield). The Regional Committees for Medical and Health Research Ethics at NTNU approved use of PBMCs from healthy adult blood donors after written informed consent (identification number 2009/2245-2). 2.5 million PBMCs were seeded per well in a 24-well plate, about 10% of which are monocytes. Monocyte-derived macrophages were generated from those by plastic adherence to the aclar substrates and maintenance in RPMI1640 (GIBCO) supplemented with 30% for 6 days (before infection) or 5% (after infection) of pooled human serum (The Blood Bank).

Transformants of the virulent *M*. *avium* clone 104 expressing CFP were used for all infection experiments, see [8] for details. Single colonies of were picked from Middlebrook 7H10 agar plates (Difco/Becton Dickinson) and grown to exponential phase (5 days) in Middlebrook 7H9 medium (Difco/Becton Dickinson) supplemented with glycerol, Tween 80, and albumin dextrose catalase. Bacteria were washed and sonicated in PBS to ensure single-cell suspensions before macrophages growing on aclar substrates were infected at a multiplicity of infection of 10. Excess bacteria were removed 4 hours post infection by washing with Hanks Balanced Salt Solution (Sigma Aldrich) and infected macrophages were left in RPMI/5% pooled human serum for two more days before examination. On average about 70% of the macrophages in microwells were infected (n = 100).

### Light microscopy

Two days post infection, macrophages were fixed using glutaraldehyde (GA, 50% EM grade, Chemi-teknik AS) in 0.15M HEPES (Sigma-Aldrich) at pH 7,2. Fixation was performed in two steps. First double strength fixative (4% GA) preheated to 37*°*C was added 1:1 directly to the growth medium of the macrophages. After 20 minutes, the solution was exchanged with normal strength fixative (2% GA) and fixation continued for 2 hours. After fixation the cells were washed in 0.3M HEPES. Subsequently fluorescence staining of DNA for nuclear visualization was performed by adding 2.5 μM Draq5 (AH diagnostics) for 10 minutes at room temperature. Confocal imaging of fixed cells was performed with a Zeiss LSM 510 Meta, by putting the film upside down in a glass bottomed dish of thickness 170μm. Cells of interest were protected from being crushed towards the confocal dish because they grew in wells and were hence lifted up 20 μm from the bottom glass. Using a 63x water objective with numerical aperture 1.2, all cells were within the accepted working distance of the objective.

### FIB/SEM sample preparation

After confocal imaging, samples were postfixed and stained for 4 hours in 1,5% potassiumferrocyanide and 2% osmium tetroxide in 0,1M cacodylate buffer. The samples were then washed 3 times in Milli-Q (MQ) water and left in MQ-water at 4*°*C over night. The subsequent day, samples were dehydrated in a graded series of 50%, 70% and 90% ethanol (15min per step on shaker), before they were *en bloc* stained in 2% uranyl acetate (UA) in 75% ethanol for 1 hour and 20 minutes. After staining, the samples were washed three times in 90% ethanol and further dehydrated 10 min in 90%, 4x15min in 100% ethanol and 2x15min in acetone. The epoxy resin used for infiltration was a mixture of 51% LX-112 epoxy resin, 27% DDSA (Dodecenyl succinic anhydride) and 22% NMA (Nadic methyl anydride), all from LADD Research Industries. 0.15ml DMP-30 (2,4,6-tris (dimethylaminomethyl) phenol, Chemi-Teknik AS) was added to 10ml of mixed epoxy resin just before use. Resin infiltration was performed in three steps of 45min, 1:2, 1:1 and 2:1 resin:acetone respectively. The samples were then left in fresh epoxy on a rotator over night. The following day, monolayer embedding was carried out using epoxy newly mixed with DMP-30 and TEM embedding moulds. The moulds were filled with resin, and the aclar substrates were placed wells-down on top, resting on the edges of the embedding moulds. The resin was polymerized at 60*°*C for 2 days, before the aclar was peeled off using tweezers. Empty epoxy extending outside the monolayer was cut off using a razor blade. The epoxy blocks were mounted on aluminum sample stubs using a drop of epoxy that was cured over night at 60*°*C. Total curing time thus became 3 days for FIB/SEM samples. Before imaging, samples were sputter coated twice at different angles with a Cressington sputter coater model 208 HR, each time with a 20 nm thick layer of platinum/palladium.

### FIB/SEM imaging

All FIB/SEM experiments were performed with a Helios NanoLab DualBeam FIB/SEM instrument from FEI Company. Cells of interest were localized using the gridded reference system on the epoxy block, and a protective platinum (Pt) layer of thickness 1 μm was deposited on the top face of the volume of interest using the gas injection system (GIS) in the FIB/SEM instrument. The ion beam was used to induce Pt deposition, at 30kV acceleration voltage and a current corresponding to a current density of 2.55 pA μm^*−*2^. The front face of the relevant well was then milled with the ion beam at 2.7 nA beam current. To avoid shadow effects and redeposition of material during imaging, trenches of about 8 μm width were milled on each side of the cell of interest with an ion beam current of 2.7 nA. The desired position of the trenches could be accurately localized by measuring distances on the corresponding confocal image of the well of interest. Finally the front face of the volume of interest was polished with an ion beam current of 0.9 nA.

FIB/SEM tomography was carried out using the Slice and View G2 software from FEI. The electron beam acceleration voltage was set to 3kV, the beam current to 0.69 nA, the resolution to 2048 *×* 1768 pixels and the dwell time to 10 μs. Images were collected in immersion mode, using the in-lens detector set to secondary electron detection. A magnification of x6500 was used for electron imaging. The number of slices was set so that the slice thickness became 35 nm, and the ion beam current was set to 0.9 nA for milling during the experiment. The milling depth was set to 3.5 μm and the milling material to Si. A protective pad of Pt with a thickness of 0.6 μm was deposited, before a fiduciary marker was etched with the ion beam. Ion beam drift correction images were collected with a resolution of 1024 *×* 884 and a dwell time of 3μs, using the Everhart-Thornley detector in secondary electron mode.

### TEM

One sample that was prepared for FIB/SEM as described above, was further processed for TEM imaging after epoxy embedding. Thin sections of 60 nm were cut using a Leica UC6 Microtome with a Diatome diamond knife. Sections were picked up on formvar coated Gilder copper slot grids. Some sections were stained in 4% UA in 50% ethanol for 12 minutes, rinsed in distilled water then counterstained in 4% lead citrate for 4 minutes, while some were imaged without further processing. TEM imaging was performed using a Philips Tecnai 12 Microscope.

### Data processing

The images collected during the FIB/SEM tomography experiment were first aligned in Fiji using StackReg or linear stack alignment with SIFT, with the transformation set to translation [51–53]. Then all images were scaled with a factor of 1.27 in y-direction using a bicubic interpolation. This scaling is necessary to obtain a realistic volume rendering, due to the geometry of the beams in the FIB/SEM instrument. Image background was subtracted with a rolling ball radius of 100 pixels, and the brightness and contrast was adjusted. After this initial processing in Fiji, the image stack was imported into Avizo (Avizo fire v 8.0, FEI Visualization Sciences Group), with a scale factor of 3.63 in z direction. The z scaling comes from the fact that the stack was collected with a slice thickness of 35nm, while the width of a pixel in x-y was 9.63nm. In Avizo, surfaces were rendered by creating labelfields and using the segmentation module. Structures of interest were traced mostly manually, because no automatic segmentation performed satisfactorily on the large and detailed dataset. Interpolation between slices was applied when appropriate. After surface generation, the number of surface triangles was reduced, and surface smoothing was performed with 10 iterations and lambda 0.6.

The z stack images collected with confocal fluorescence microscopy (with DIC overlay) were adjusted for brightness and contrast in all channels, and a Gaussian blur filter of radius 1 was applied in Fiji to reduce noise. Following this the stack was imported into Avizo, with a scale factor of 7.25 in z direction (pixel size in x-y was 120nm, while images from the z stack were collected with a distance of 870nm). Bacteria and nucleus were then surface rendered using Isosurface Rendering in Avizo, with a threshold of 43.2 and 60.7 respectively.

Images from FIB/SEM tomography and confocal fluorescence microscopy of the same cell were correlated in Avizo, based on features of the aclar substrate. All relevant images and stacks (ion image stack taken for drift correction purposes during the slice and view experiment, confocal z-stack, FIB/SEM tomography stack and overview image from the tomography surface) were first imported into Avizo with the correct nm/pixel values. The ion images were aligned using the linear stack alignment with SIFT in Fiji [51, 52] before import, and the z voxel size was set to 0.00001 upon import to Avizo since these images all originate from the same plane. The image corresponding to the bottom of the well in the confocal z stack was identified, and this image was placed at z = 0 in the coordinate system of Avizo. In the 0 plane, the drift correction images were then aligned with this confocal image. As a result, a connection between electron images and light microscopy images was established, with the depth of the well correctly placed. The FIB/SEM tomography stack was then placed with the first image in the plane of the first drift correction image, and the last image in the plane of the last. The overview image of the tomography surface taken just before the experiment started was aligned with the first drift correction image, at the correct z and xy position. The correct xy position of the FIB/SEM tomography stack was then found by overlay of the first image with the latter. After this procedure, the volume from the FIB/SEM tomography experiment was correctly placed within the confocal z stack volume, and corresponding images in all planes could be identified.

## Supporting Information

S1 Fig Geometry of the FIB/SEM instrument, and imaging at a flat surface or at a sample edge. In the Dualbeam instrument from FEI used in this study, the electron beam and the ion beam are positioned at 52° tilt compared to each other. During FIB/SEM tomography experiments, the ion beam mills off successive slices at a gracing incidence, while the electron beam images each appearing surface of interest at an angle of 52° compared to the image plane. a) Situation where the region of interest (red) is at a sample edge, and milling and imaging can be started immediately. b) The region of interest is in the middle of a flat surface, and a lot of material needs to be removed before the electron beam can access the whole surface to be imaged. Even more material needs to be removed to avoid shadow effects, because secondary electrons emitted close to a trench wall can be absorbed and will never reach the detector. Trench milling is often done with high ion beam currents to reduce milling time, but this is potentially harmful for the sample.
(TIF)

S2 Fig Quality of the master and the moulding process. The master, aclar and final epoxy block were characterized by SEM imaging. SEM imaging was performed at a 52° tilt angle in order to better observe edge profiles. a) SEM micrograph of a corner of a protruding square on the silicon master. b) The corresponding well on a patterned aclar substrate. c) SEM micrograph displaying the final small epoxy block after embedding. All features are conserved throughout the moulding process, exemplified by the small notch indicated by the arrow on all images. After epoxy embedding, the microwell geometry enabled access to a large number of cells at these straight, vertical sample edges, which greatly facilitated FIB/SEM tomography when compared to flat monolayers. Scale bar 20 μm.
(TIF)

S3 Fig SEM imaging at low and high kV. a) and b) are SEM micrographs collected from the surface of the same epoxy block containing embedded macrophages. a) SEM micrograph collected with an acceleration voltage of 3kV. b) SEM micrograph collected with an acceleration voltage of 15kV. The high acceleration voltage used in in b) allows observing the shape of cells just below the surface, while 3kV imaging (a) only shows the surface topography of the block. Scale bar 100μm.
(TIF)

S4 Fig SEM vs TEM Comparison. Two samples with primary human macrophages grown on aclar substrates were prepared in parallel, i.e. with identical conditions for cell growth and sample preparation. One was imaged with SEM during a FIB/SEM tomography experiment (column 1), while the other was sectioned and imaged with TEM, both without (column 2) and with additional staining on the thin sections (column 3). Mitochondria and Golgi are shown as a comparison of the level of detail caught by the two imaging techniques. SEM images all originate from the same tomography data, meaning detail images are cropped out from a larger image of 20 μm x 20 μm. The magnification for the TEM images varies. Scale bars row 1: 5μm, row 2–3: 1μm
(TIF)

S1 Video Video going through all images from the FIB/SEM tomography image stack (in the x-z and y-z planes), before the generation of surfaces from the stack is illustrated. Clearly resolved membranes were used to determine boundaries of the nucleus (blue), a vesicle (yellow) and bacteria (red).
(MP4)
