# Supplementary figures and images for: Seeing a Mycobacterium-Infected Cell in Nanoscale 3D: Correlative Imaging by Light Microscopy and FIB/SEM Tomography
Source: PLoS One. 2015 Sep 25;10(9):e0134644. doi: 10.1371/journal.pone.0134644 (PMC4583302; doi:10.1371/journal.pone.0134644)

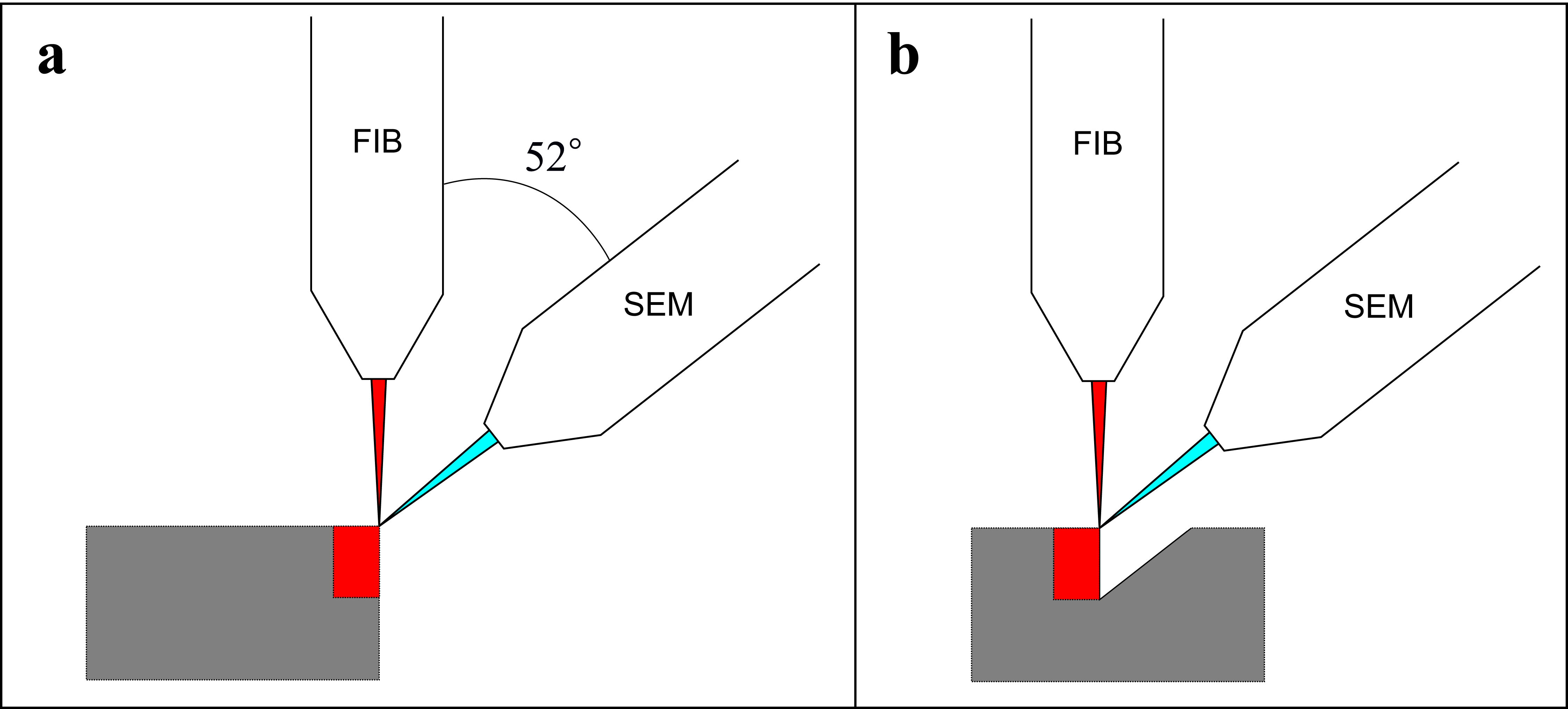

Supplement: S1 Fig — In the Dualbeam instrument from FEI used in this study, the electron beam and the ion beam are positioned at 52° tilt compared to each other. During FIB/SEM tomography experiments, the ion beam mills off successive slices at a gracing incidence, while the electron beam images each appearing surface of interest at an angle of 52° compared to the image plane. a) Situation where the region of interest (red) is at a sample edge, and milling and imaging can be started immediately. b) The region of interest is in the middle of a flat surface, and a lot of material needs to be removed before the electron beam can access the whole surface to be imaged. Even more material needs to be removed to avoid shadow effects, because secondary electrons emitted close to a trench wall can be absorbed and will never reach the detector. Trench milling is often done with high ion beam currents to reduce milling time, but this is potentially harmful for the sample. (TIF) [file pone.0134644.s001.tif]

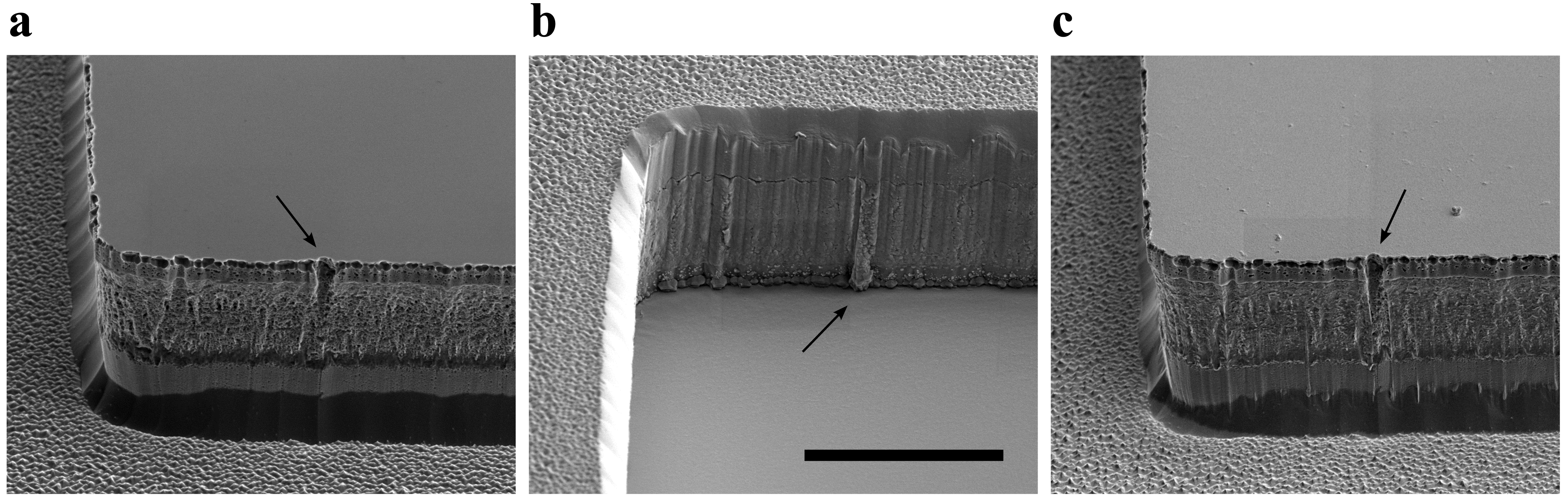

Supplement: S2 Fig — The master, aclar and final epoxy block were characterized by SEM imaging. SEM imaging was performed at a 52° tilt angle in order to better observe edge profiles. a) SEM micrograph of a corner of a protruding square on the silicon master. b) The corresponding well on a patterned aclar substrate. c) SEM micrograph displaying the final small epoxy block after embedding. All features are conserved throughout the moulding process, exemplified by the small notch indicated by the arrow on all images. After epoxy embedding, the microwell geometry enabled access to a large number of cells at these straight, vertical sample edges, which greatly facilitated FIB/SEM tomography when compared to flat monolayers. Scale bar 20 μm. (TIF) [file pone.0134644.s002.tif]

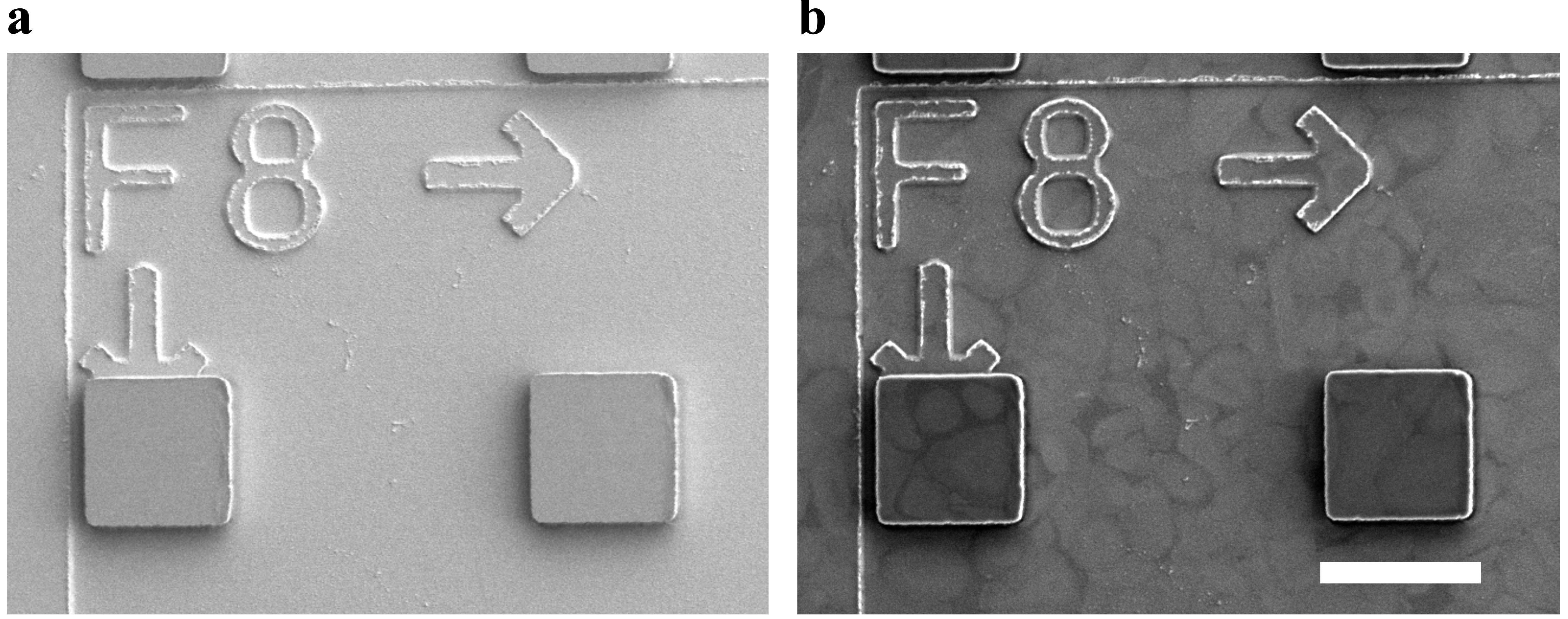

Supplement: S3 Fig — a) and b) are SEM micrographs collected from the surface of the same epoxy block containing embedded macrophages. a) SEM micrograph collected with an acceleration voltage of 3kV. b) SEM micrograph collected with an acceleration voltage of 15kV. The high acceleration voltage used in in b) allows observing the shape of cells just below the surface, while 3kV imaging (a) only shows the surface topography of the block. Scale bar 100μm. (TIF) [file pone.0134644.s003.tif]

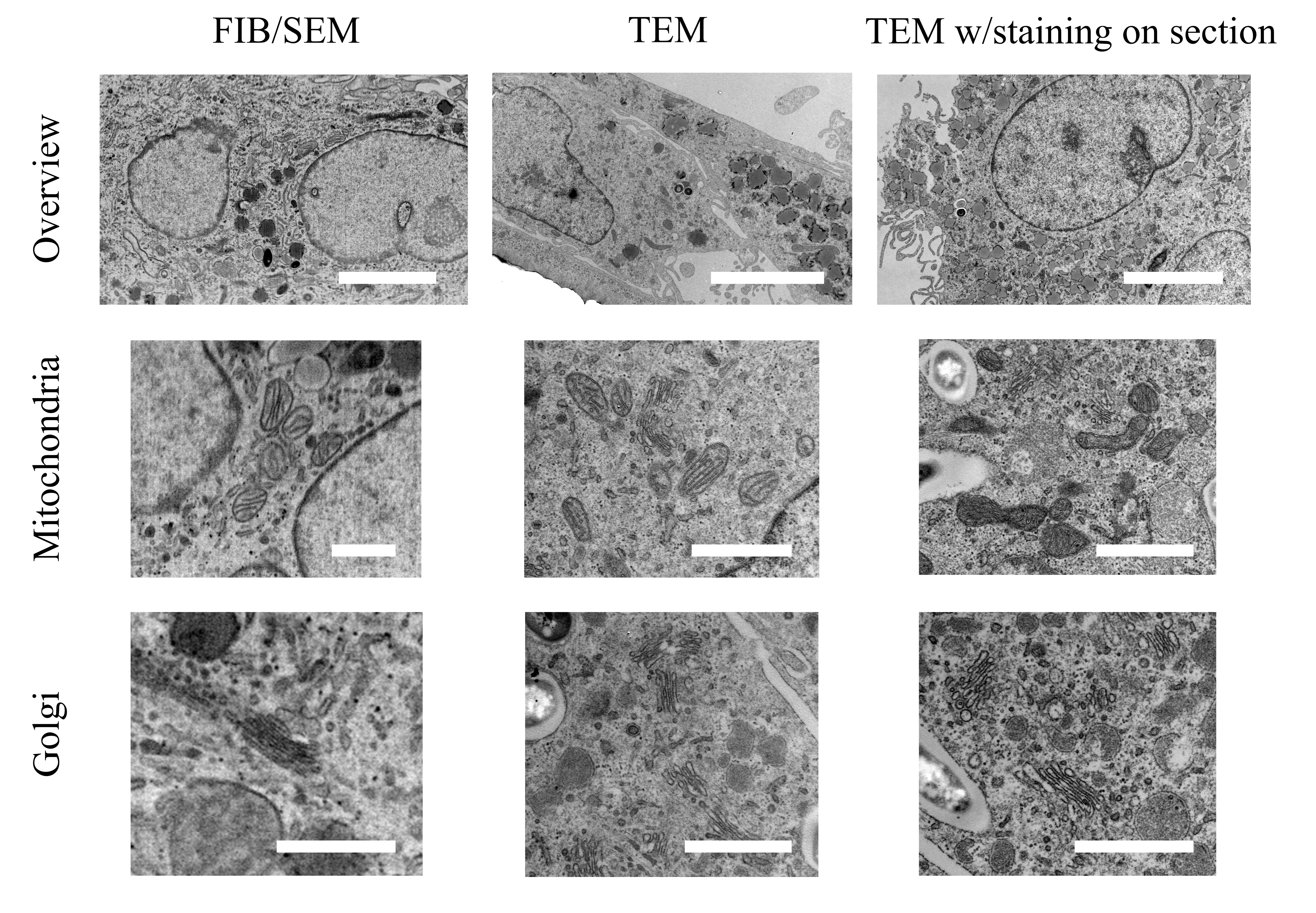

Supplement: S4 Fig — Two samples with primary human macrophages grown on aclar substrates were prepared in parallel, i.e. with identical conditions for cell growth and sample preparation. One was imaged with SEM during a FIB/SEM tomography experiment (column 1), while the other was sectioned and imaged with TEM, both without (column 2) and with additional staining on the thin sections (column 3). Mitochondria and Golgi are shown as a comparison of the level of detail caught by the two imaging techniques. SEM images all originate from the same tomography data, meaning detail images are cropped out from a larger image of 20 μm x 20 μm. The magnification for the TEM images varies. Scale bars row 1: 5μm, row 2–3: 1μm (TIF) [file pone.0134644.s004.tif]
